# Fractional Excretion of Urate for Diuresis Management in Heart Failure and Cardiorenal Syndrome

**DOI:** 10.1016/j.jaccas.2020.12.035

**Published:** 2021-04-07

**Authors:** Amnon A. Berger, Thomas L. Mawson, Andre Dejam

**Affiliations:** a Department of Anesthesia, Critical Care and Pain Medicine, Beth Israel Deaconess Medical Center and Harvard Medical School, Boston, Massachusetts, USA; b Internal Medicine, Beth Israel Deaconess Medical Center and Harvard Medical School, Boston, Massachusetts, USA; c Division of Cardiovascular Medicine, Beth Israel Deaconess Medical Center and Harvard Medical School, Boston, Massachusetts, USA

**Keywords:** CRS, diuresis, FeNa, FeUa, uric acid, volume overload, AKI, acute kidney injury, AS, aortic stenosis, BNP, brain natriuretic peptide, CKD, chronic kidney disease, COPD, chronic obstructive pulmonary disease, CXR, chest x-ray, DOE, dyspnea on exertion, ECFV, extracellular fluid volume, ED, emergency department, EF, ejection fraction, FeUa, fractional excretion of uric acid, HF, heart failure, HFrEF, heart failure with reduced ejection fraction, ILD, interstitial lung disease, IV, intravenous, PE, physical examination, SOB, shortness of breath

## Abstract

Most heart failure hospitalizations are due to volume overload; however, it is not easily evaluated by physical examination. Avoidance of diuresis in patients with fluid overload to avoid acute kidney injury increases morbidity in heart failure. We hypothesize that fractional excretion of urate can be used to guide diuresis. (**Level of Difficulty: Advanced.**)

## Central Illustration

**Figure:**
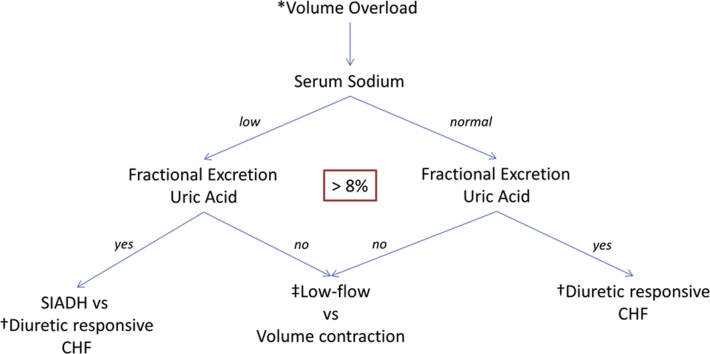

Hospitalizations for HF are frequently due to symptomatic volume expansion. Strategies to reduce volume overload reduce hospitalizations and improve quality of life. Biomarker approaches (eg, brain natriuretic peptide [BNP]) as well as physical examination (PE) show limited sensitivity and specificity. Rise of creatinine is frequently encountered when patients are diuresed, left only partially decongested with increased morbidity due to fear of worsening acute kidney injury (AKI) (1, 2, 3).

**Learning Objectives**

• To identify the challenges of volume status assessment in patients with HF and the accompanied difficulty in determining diuretic management in these patients.
• To understand the calculation of FeUa and recognize the conditions limiting this calculation.
• To apply FeUa measurements as an adjunct to diuretic management in patients presenting in suspected heart failure with volume overload.

Fractional urine excretion of uric acid (FeUa) allows accurate differentiation of hyponatremia by volume status, despite diuretic use. Elevated FeUa identifies, with high sensitivity and specificity, hyponatremic patients with volume expansion due to syndrome of inappropriate antidiuretic hormone. Low FeUa was described in renal salt wasting and hypovolemia (4, 5, 6, 7).

**Figure 1:**
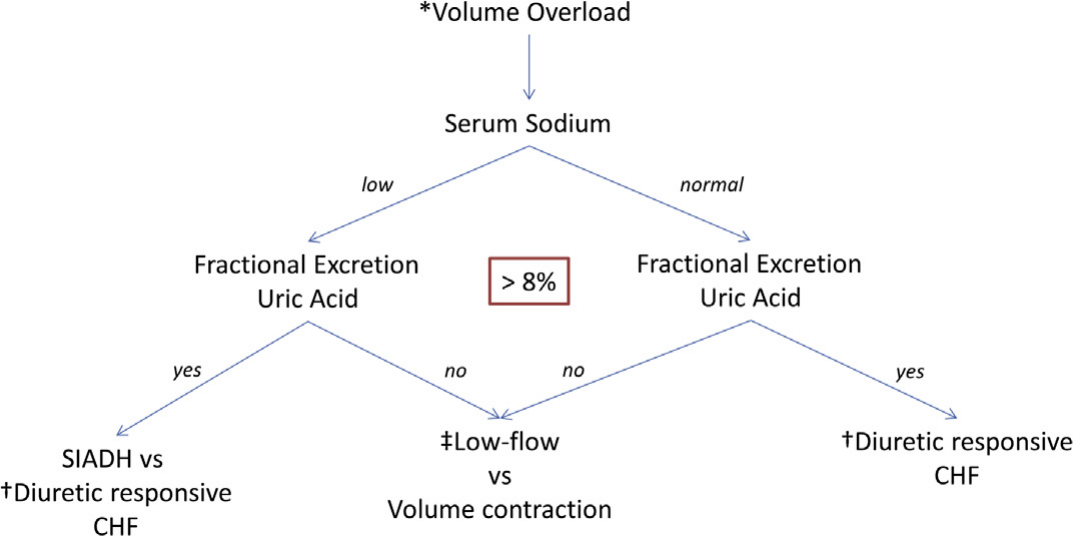
Proposed Assessment of Volume Overload Using Fractional Excretion of Uric Acid We hypothesize that fractional excretion of uric acid >8% indicates loop diuretic responsive fluid overload with low probability of diuretic-induced kidney injury with continued diuresis. ∗Volume overload is defined by clinically determined expansion of interstitial volume (leg swelling, ascites, pleural effusion) or intravascular volume (right atrial pressure elevation by JVD/inferior vena cava plethora using ultrasound, left atrial pressure elevation [lung ultrasound, crackles] and hemodynamic congestion [B-type natriuretic peptide elevation]). †Diuretic-responsive CHF: expansion of interstitial/intravascular fluid space in setting of elevated ventricular transmural pressure due to abnormal cardiac function, responsive to diuretic agents with low probability of inducing acute kidney injury. ‡Low-flow state: decreased renal blood flow due to low cardiac output (cardiac index <2.5 l/min/m^2^) or cold extremities/lactate elevation. CHF = congestive heart failure; SIADH **=** syndrome of inappropriate anti-diuretic hormone.

We hypothesize that FeUa >8% identifies patients that can be diuresed with low risk of AKI (Figure 1), as presented by 6 cases (Table 1), in all FeUa-guided diuretic management where PE and BNP were inconclusive.

**Table 1:** Demographic Data and Cardiac History

Patient	Age, y	Sex	Ethnicity	Known HF	EF	Admit BNP	Diuretic Agents Pre-Admission	Diuretic Agents During Admission
#1	64	Male	White	No	48%	2,162	None	IV furosemide, PO torsemide
#2	78	Female	White	No	35%	30,686	None	IV furosemide, PO torsemide
#3	83	Female	White	Diastolic	60%	590	PO Furosemide	IV furosemide
#4	83	Male	White	Systolic	50%	3,714	None	IV furosemide
#5	77	Male	White	Systolic	42%	8,114	None	IV furosemide
#6	50	Male	Black	Systolic	20%	25,116	None	IV furosemide, PO torsemide

Basic demographic data of the patients in this case series, including age, sex, and ethnicity, alongside cardiac history. 2 patients had no history of heart failure (HF), 1 had known HF with preserved ejection fraction (EF), and 3 were previously diagnosed with HF with reduced EF. Only patient 3 was taking diuretic agents prior to this admission. All patients were treated with intravenous furosemide; 3 patients (#1, #2, and #6) were also treated with oral torsemide.

BNP = B-type natriuretic peptide.

## Case Presentations

Patient #1 is a 64-year-old man with history of smoking, peripheral artery disease, type 2 diabetes mellitus, and chronic obstructive pulmonary disease (COPD). He presented to our emergency department (ED) due to month-long escalating shortness of breath (SOB). He was recently treated for presumed worsening COPD with inhalers and steroids and was referred to the ED when his SOB failed to resolve. His PE was noticeable for atrial tachycardia with heart rates of 110 to 120 beats/min and hypoxia with oxygen saturation of 80% in room air. A chest x-ray (CXR) revealed mild pulmonary edema and small pleural effusions, but there were no other signs of fluid overload; he appeared euvolemic on examination.

Transthoracic echocardiogram revealed a newly reduced ejection fraction (EF) (48%). He was treated with intravenous diuretic agents and improved clinically. He remained euvolemic on examination. However, his atrial tachycardia continued, and he had not returned to his respiratory baseline. He developed asymptomatic hyponatremia (nadir 127 mEq/l). Serum creatinine and bicarbonate remained at baseline. It was unclear if he was being overtreated with diuretic agents, causing his hyponatremia, or undertreated with continued expansion of his extracellular fluid volume (ECFV). There was no clinical evidence to suggest he was hypervolemic, aside from increased SOB on bending forward (he was not orthopneic). A bedside ultrasound was performed, which showed an enlarged 2.5-cm inferior vena cava with >50% collapsibility, but with a poor image quality. A fractional excretion of uric acid (FeUa) was calculated as 11%. Given his high FeUa and bedside ultrasound, intravenous (IV) furosemide was restarted. Diuretic agents were continued for 4 days with symptomatic improvement. He was able to ambulate without dyspnea, he was not SOB with bending, and his heart rate normalized gradually (as seen on telemetry) until his atrial tachycardia abated and was replaced with normal sinus rhythm. Serum sodium improved (peak 131 mEq/l), and diuretic agents were stopped when it down-trended; then, FeUa was 5.46% and inferior vena cava <2.1 cm with >50% collapse on ultrasound. He continues to do well as an outpatient with no recurrence of SOB or hyponatremia.

Patient #2, a 78-year-old woman, was transferred to our intensive care unit in multifactorial respiratory failure from multifocal pneumonia, new heart failure with reduced ejection fraction (HFrEF) (EF 35%), and severe (previously mild to moderate) aortic stenosis (AS). She was treated with diuretic agents and care was continued on the medicine floor; diuretic agents were held for several days under the impression of euvolemia and preload-dependency given AS. She continued to require oxygen with significant orthopnea. A right heart catheterization showed significant fluid overload; thus, continuous IV furosemide was restarted, with good response, including slowly weaning from oxygen and clinical improvement. Creatinine rose to 1.2 mg/dL from 1.0 mg/dL after 5 days of IV diuretic agents, which is concerning for over-treatment. FeUa was 6.94%; this prompted transition to maintenance oral diuresis, and creatinine normalized within 3 days.

Patient #3, an 83-year-old woman, presented with clinically overt HF with preserved EF exacerbation. Diuretic agents were started in the ED, and an FeUa of 9.6% was calculated. She continued IV diuretic agents until she improved clinically and was discharged with oral diuretic agents without worsening of her kidney function.

Patient #4, an 83-year-old man, was transferred to our cardiac service for consideration of coronary angiogram indicating chest pain a week prior and ongoing dyspnea on exertion (DOE). He had known coronary artery disease with previous interventions, HFrEF (EF 50%), and stage III chronic kidney disease (CKD). It was unclear whether his DOE is an anginal-equivalent. FeUa was 14.0%. He was started on IV diuretic agents and improved quickly; his DOE was relieved completely, and his chest pain did not recur. Angiography was not performed as his symptoms resolved. He was discharged home with continued oral diuretic agents.

Patient #5, a 73-year-old man, had known HFrEF, CKD, severe AS, and interstitial lung disease (ILD). He presented with 8 months of progressive SOB and new oxygen requirement at rest, which could have been attributed to AS, ILD, or fluid overload. He was euvolemic on examination. He was started on IV diuretic agents while FeUa was pending. In 2 days of diuretic agents, creatinine rose mildly, without clinical improvement. His FeUa returned at 1.04%, unlikely for fluid overloaded, and diuretic agents were stopped. He experienced no clinical change, and his oxygen requirement was deemed secondary to his AS and ILD.

Patient #6, a 50-year-old man, has severe HFrEF (EF 20%), COPD, and stage III CKD who presented with SOB and cough after completing treatment for community-acquired pneumonia. His history and CXR suggested a loculated effusion. He was treated with broad-spectrum antibiotics and a chest tube that drained borderline transudative-exudative fluid. The FeUa was calculated to be 8.12%. Two days later, his SOB persisted and CXR showed bilateral pleural effusions. He was started on IV diuretic agents, improved promptly, and was discharged home 2 days later with continued oral diuretic agents.

## Discussion

There is a small body of literature evaluating the utility of FeUa in determining volume status in hyponatremic patients (4,6,8, 9, 10). FeUa has been used to identify fluid responsiveness in kidney transplant patients previously (9). It has been shown that the utility of FeUa to diagnose volume expansion does not change depending on urine flow or use of loop diuretic agents (unlike FeNa), but is mostly a function of changes in ECFV (4,5,11). We leveraged the utility of FeUa in different ECFV states and present for the first time observations supporting the use of FeUa to direct diuretic use in congestive HF.

We presented 6 cases where determining the fluid status of patients was difficult clinically, biochemically, and even using bedside ultrasound. In each of these cases, FeUa (Table 2) proved to be a valuable adjunct to our clinical evaluation of patients.

**Table 2:** Laboratory Findings

Patient	S. Creatinine, mg/dL	S. Sodium, mEq/L	S. Uric Acid, mg/dL	S. Urea, mg/dL	S. Osm, mOsm/kg	U. Creatinine, mg/dL	U. Sodium, mEq/L	U. Uric Acid, mg/dL	U. Urea, mg/dL	U. Osm, mOsm/kg	FeNa, %	FeUa, %	FeUrea, %
#1	0.8	127	5.4	23	274	81	34	59.9	918	492	0.26	10.96	39.42
#1	1.1	130	8.1	26	270	85	44	34.2	597	426	0.44	5.46	29.71
#2	1.2	137	4.6	19	282	59	60	15.7		338	0.89	6.94	
#3	0.6	131	4.2	13		35	49	23.5		278	0.64	9.59	
#4	1.9	140	5.2	37	300	71	106	27.1	814	572	2.03	13.95	58.87
#4	2.3	136		52		147	30			552	0.35		
#5	1.6	141	6.2	29	287	275	62	11.1		311	0.26	1.04	
#6	2.1	137	6.9	28		314	35	83.8	1,506	871	0.17	8.12	35.97

Laboratory data for patients. Some patients may have had laboratory tests drawn more than once. Data includes serum (“S”) creatinine, sodium, uric acid, urea, osmolality (osm), and urine (“U”) creatinine, uric acid, urea and osmolality. Fractional excretion of sodium (FeNa), fractional excretion of uric acid (FeUa), and fractional excretion of urea (FeUrea) were calculated from these data.

The exact cutoff for FeUa to indicate volume expansion is unclear, and research is likely needed to set that boundary. Previous studies used different cutoffs for FeUa in an adult population, such as 8%, 10%, and 12% in other disease states (4). More recent evidence point to normal FeUa close to 5%, with 10% to 11% representing the 95th percentile (12). In our small sample, 8% to 10% seems to represent a threshold for action, and FeUa <5% represents high risk for AKI and unlikely hypovolemia. It may also be hindered by uricosuric drugs and losartan (which our patients were not taking).

Our study has many limitations. It is a small case series, lacking randomization or control for confounding. There was no control group, and the same group of physicians cared for patients. Nonetheless, it presents supporting evidence that FeUa could be an adjunct to established tools in the management of diuretic therapy, does not require specific skills (such as ultrasound), and is unaffected by concurrent diuresis (unlike FeNa).

## Conclusions

We present a string of clinical observations to support the hypothesis that FeUa can identify patients with hard to assess volume status that are responsive to diuresis with low risk of AKI. FeUa served as a valuable tool to guide initiation, dosing, and termination of diuretic therapy.

## Funding Support and Author Disclosures

All named authors meet the International Committee of Medical Journal Editors (ICMJE) criteria for authorship for this paper, take responsibility for the integrity of the work as a whole, and have given their approval for this version to be published. The authors have reported that they have no relationships relevant to the contents of this paper to disclose.

## References

[1] Kurmani S. (2017). Squire I. Acute heart failure: definition, classification and epidemiology. Curr Heart Fail Rep.

[2] Bleumink G.S., Knetsch A.M., Sturkenboom M.C.J.M. (2004). Quantifying the heart failure epidemic: Prevalence, incidence rate, lifetime risk and prognosis of heart failure - The Rotterdam Study. Eur Heart J.

[3] Farmakis D., Parissis J., Lekakis J., Filippatos G. (2015). Acute heart failure: epidemiology, risk factors, and prevention. Rev Española Cardiol (English Ed.

[4] Fenske W., Störk S., Koschker A.C. (2008). Value of fractional uric acid excretion in differential diagnosis of hyponatremic patients on diuretics. J Clin Endocrinol Metab.

[5] Steinman T.I. (2008). Can the fractional excretion of uric acid distinguish the etiology of hyponatremia in patients taking diuretics?. Nat Clin Pract Endocrinol Metab.

[6] Imbriano L.J., Mattana J., Drakakis J., Maesaka J.K. (2016). Identifying different causes of hyponatremia with fractional excretion of uric acid. Am J Med Sci.

[7] Spasovski G., Vanholder R., Allolio B. (2014). Clinical practice guideline on diagnosis and treatment of hyponatraemia. Eur J Endocrinol.

[8] Kosmadakis G., Viskaduraki M., Michail S. (2009). The validity of fractional excretion of uric acid in the diagnosis of acute kidney injury due to decreased kidney perfusion. Am J Kidney Dis.

[9] Choi J.W., Park J.S., Koo T.Y., Lee C.H., Kang C.M., Kim G.H. (2013). Fractional excretion of uric acid as a predictor for saline responsiveness in long-term kidney transplant patients. Kidney Blood Press Res.

[10] Maesaka J.K., Miyawaki N., Palaia T., Fishbane S., Durham J.H.C. (2007). Renal salt wasting without cerebral disease: diagnostic value of urate determinations in hyponatremia. Kidney Int.

[11] Maesaka J.K., Fishbane S. (1998). Regulation of renal urate excretion: a critical review. Am J Kidney Dis.

[12] Narang R.K., Vincent Z., Phipps-Green A., Stamp L.K., Merriman T.R., Dalbeth N. (2019). Population-specific factors associated with fractional excretion of uric acid. Arthritis Res Ther.

